# Effectiveness of Application of Oral Regimen, Practicing Oral Health, Health Education, Observation (APHO) Nursing Intervention in Preventing and Managing Oral Mucositis in Children Undergoing Chemotherapy: An Interventional Study in Central India

**DOI:** 10.7759/cureus.40902

**Published:** 2023-06-24

**Authors:** Tejaswee Lohakare, Darshana Kumari, Mayur B Wanjari, Archana Maurya, Bibin Kurian, Khushbu M Meshram

**Affiliations:** 1 Department of Child Health Nursing, Smt. Radhikabai Meghe Memorial College of Nursing, Datta Meghe Institute of Higher Education and Research, Wardha, IND; 2 Department of Research and Development, Jawaharlal Nehru Medical College, Datta Meghe Institute of Higher Education and Research, Wardha, IND

**Keywords:** chemotherapy, apho nursing intervention, oral mucositis, acute lymphoblastic leukemia, cancer

## Abstract

Background

The incidence of childhood cancer is rising worldwide, with acute lymphoblastic leukemia being the most commonly diagnosed form. These young patients are immunocompromised and susceptible to infections. In addition, chemotherapy and radiation therapy often cause oral mucositis as a side effect. Given these challenges, nurses play a crucial role in delivering special care to these vulnerable children.

Material and methods

A study design utilizing a one-group pretest-posttest approach was implemented on 45 children, aged one to 12 years, who were undergoing chemotherapy and experiencing oral mucositis. Demographic information, including age, gender, diagnosis, chemotherapy cycle, and length of therapy, was collected. A customized intervention, which involved the application of an oral regimen, oral health practices, health education for caregivers, and observation, was administered by nursing staff using the APHO (Application of an Oral Regimen, Practicing Oral Health, Health Education to Caregivers, and Observation) protocol for a period of seven days. Both pre-intervention and post-intervention assessments were conducted to determine the effectiveness of the APHO nursing intervention. The assessment tool used to assess the effectiveness of APHO intervention was using a standardized scale given by the World Health Organization grading scale for oral mucositis.

Result

An analysis was conducted utilizing both descriptive and inferential statistics. Before the intervention, those children in grade 2 were in grade 1 after the intervention, and the children in grade 1 before the intervention were in grade 0 after the intervention. The health education provided to the caregivers was significantly effective. Notably, there was a significant decrease in the oral mucositis grade of the children by one level in all instances, indicating that the APHO nursing intervention was remarkably effective. Thus, promoting the use of APHO nursing intervention can lead to positive outcomes in children afflicted with oral mucositis.

Conclusion

APHO nursing intervention has shown remarkable effectiveness among children with mucositis. As a result, promoting continuous APHO intervention in children with mucositis can help enhance in prevention and management.

## Introduction

The use of intensive regimens in treating paediatric cancer has led to a significant increase in patient survival in recent years. However, these treatments are also associated with a rise in harmful effects, including mucositis (oral cavity inflammation), which can have a substantial impact on treatment efficacy [1].

Oral mucositis (OM) is a common side effect of chemotherapy, head and neck radiation, and targeted therapy, affecting around 75% of high-risk patients [2]. Chemotherapy is the primary treatment for patients with acute lymphoblastic leukaemia (ALL) [3], but OM can cause excruciating discomfort and difficulty in consuming food and liquids, necessitating the use of narcotic analgesics, hospitalization, and supplemental nutrition [4]. These problems can disrupt cancer therapy and lower survival rates, especially in immunocompromised children who are at a high risk of developing sepsis if bacteria enter the ulcers [5].

Oral mucositis is one of the most common side effects of chemotherapy among cancer patients, affecting 52 to 80% of children [6]. It has a crippling impact during chemotherapy, with a worse effect on nutritional, physical, and psychological health [7]. While there are some effective preventive measures, ulcerative oral mucositis can be expensive for healthcare systems.

Oral mucositis is a frequent and unpleasant side effect of chemotherapy in children with cancer that reduces quality of life (QoL) and treatment adherence. Cancer and its treatment can lead to significant dysbiosis in the oral microbiota, marked by decreased richness and variety [8].

To assess and categorize children according to their grades, the World Health Organisation (WHO) has introduced a standardized grading scale for mucositis. The APHO (Application of an Oral Regimen, Practicing Oral Health, Health Education to Caregivers, and Observation) nursing intervention provides a provision for assessing efficacy and ruling out improvements in mucositis.

## Materials and methods

Study setting and design and data collection

The interventional study was carried out at a cancer hospital in Nagpur city from January 13, 2023, to March 28, 2023. A purposive sampling technique was used to obtain 45 children with oral mucositis. An experimental research design was employed with a one-group pre-test post-test approach. The study included children between the ages of one to 12 years undergoing chemotherapy with grade 1 and grade 2 oral mucositis. Children receiving radiation therapy, biotherapy, and immunotherapy were excluded from the study.

Data collection and APHO nursing intervention

The standardized scale given by the World Health Organisation grading scale for oral mucositis was used to assess the children and categorize them according to the grade of mucositis before intervention. APHO nursing intervention was then given to the children, and on the eighth day, post-assessment was conducted to identify the effectiveness of APHO nursing intervention and the status of oral mucositis.

Description of APHO nursing intervention

APHO nursing intervention aims to provide oral care through nursing intervention to heal oral mucositis. The intervention includes the assessment of oral regimen application and practicing oral health using a checklist. A structured questionnaire is used to evaluate health education related to the prevention of oral mucositis. Observations are also made using the WHO grading scale for oral mucositis to assess the improvement in the condition of the oral mucosa among children undergoing chemotherapy. Post-intervention assessment is performed on the eighth day (Table 1).

**Table 1:** Implementation of APHO nursing intervention (days) APHO: Application of an Oral Regimen, Practicing Oral Health, Health Education to Caregivers, and Observation

		Study Period							
		Day 1	Day 2	Day 3	Day 4	Day 5	Day 6	Day 7	Day 8
Pre-Assessment		X							
APHO Nursing Intervention									
Application of oral regimen		X	X	X	X	X	X	X	
Practicing oral health		X	X	X	X	X	X	X	
Health Education	Pretest	X							
	Posttest								X
Observation		X	X	X	X	X	X	X	X
Post- Assessment									X

Statistical analysis

The collected data was analyzed using the IBM SPSS 25.0 version (IBM Corp., Armonk, NY, USA). Descriptive statistics were used to obtain results, and the chi-square test was used to determine any associations with demographic variables.

## Results

Demographic variables

This section pertains to children who have oral mucositis and are undergoing chemotherapy. The data collected includes demographic variables such as age, sex, diagnosis, cycle of chemotherapy, and therapy length (Table 2).

**Table 2:** Percentage-wise distribution of children with oral mucositis undergoing chemotherapy.

Demographic Variable	No. of Children	Percentage
Age in years		
1 to 3 Years	4	8.9%
4 to 6 Years	21	46.7%
7 to 9 Years	10	22.2%
10 to12 Years	10	22.2%
Gender		
Male	27	60%
Female	18	40%
Diagnosis		
Acute Lymphoblastic Leukemia	21	46.7%
Acute Myeloid Leukemia	2	4.4%
Ewing Sarcoma	1	2.2%
Germ Cell Tumour	1	2.2%
Hodgkin’s Lymphoma	6	13.3%
Medulloblastoma	1	2.2%
Neuroblastoma	1	2.2%
Non- Hodgkin’s Lymphoma	5	11.1%
Osteogenic Sarcoma	4	8.9%
Rhabdomyosarcoma	1	2.2%
Wilm’s tumour	2	4.4%
Cycle of Chemotherapy		
1^st^ cycle	16	35.6%
2^nd^ Cycle	12	26.7%
3^rd^ Cycle	8	17.8%
4^th^ Cycle	2	4.4%
5^th^ Cycle	7	15.6%
Therapy Length		
1 day	0	0%
2 days	0	0%
3 days	1	2.2%
4 days	1	2.2%
5 days	31	68.9%
6 days	10	22.2%
7 days	0	0%
8 days	2	4.4%

Assessment of APHO nursing intervention

The nursing intervention developed by the APHO to achieve a positive outcome for oral mucositis is classified into four grades: normal (0), mild (1), moderate (2), severe (3), and extreme (4). For this particular study, only grade 1 and grade 2 were considered. Prior to the intervention, 16 (35.6%) children were classified as grade 1 and 29 (64.4%) were classified as grade 2. After the intervention, oral mucositis was successfully treated and 16 (35.6%) children were classified as normal grade 0, while 29 (64.4%) were classified as mild grade 1 (Table 3).

**Table 3:** Assessment of before and after intervention children with oral mucositis according to WHO grading scale.

WHO grading Scale	Range	Before Intervention		After Intervention	
		Frequency	Percentage	Frequency	Percentage
Normal	0	0	0	16	35.6%
Grade 1	1	16	35.6%	29	64.4%
Grade 2	2	29	64.4%	0	0
Grade 3	3	0	0	0	0
Grade 4	4	0	0	0	0

Effectiveness of APHO nursing intervention on children with oral mucositis undergoing chemotherapy

**Figure 1:**
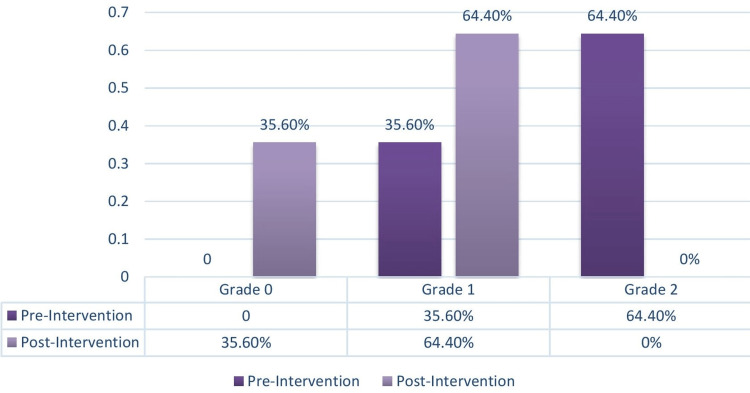
Effectiveness of intervention

The pre-intervention scores revealed that 16 children (35.6%) were in grade 1 and 29 children (64.4%) were in grade 2. Following the intervention, the children who were initially in grade 1 recovered and were reclassified as grade 0. Similarly, the children in grade 2 improved and were reclassified as grade 1. This demonstrates the effectiveness of the intervention, as shown in Figure 1.

Effectiveness of health education to the caregiver of children undergoing chemotherapy before and after was seen as significant with a p-value of 0.001 and t-value of 12.02 (Table 4). 

**Table 4:** Effectiveness of health education to the caregiver of children undergoing chemotherapy

Effectiveness of health education	Mean	SD	Mean Difference	t- value	p-value
Before	2.68	0.92	1.56±0.12	12.02	0.001
After	4.24	0.80			

Association of the findings with demographical variables

There were no significant differences in the age, sex and diagnosis, cycle of chemotherapy and therapy length (Table 5).

**Table 5:** Association of APHO nursing intervention with demographic variables. APHO: Application of an Oral Regimen, Practicing Oral Health, Health Education to Caregivers, and Observation

Demographic Variable	Normal	Grade 1	Grade 2	Total	Chi-square	Df	P-Value
Age in years							
1 to 3 Years	0	4	0	4	5.34	3	0.148 NS
4 to 6 Years	6	15	0	21			
7 to 9 Years	4	6	0	10			
10 to12 Years	6	4	0	10			
Gender							
Male	9	18	0	27	0.145	1	0.703 NS
Female	7	11	0	18			
Diagnosis							
Acute Lymphoblastic Leukemia	5	16	0	21	13.31	10	0.206 NS
Acute Myeloid Leukemia	2	0	0	2			
Ewing Sarcoma	1	0	0	1			
Germ Cell Tumour	0	1	0	1			
Hodgkin’s Lymphoma	3	3	0	6			
Medulloblastoma	1	0	0	1			
Neuroblastoma	0	1	0	1			
Non- Hodgkin’s Lymphoma	2	3	0	5			
Osteogenic Sarcoma	1	3	0	4			
Rhabdomyosarcoma	1	0	0	1			
Wilm’s tumour	0	2	0	2			
Cycle of chemotherapy							
1^st^ cycle	6	10	0	16	2.96	4	0.56 NS
2^nd^ Cycle	4	8	0	12			
3^rd^ Cycle	2	6	0	8			
4^th^ Cycle	0	2	0	2			
5^th^ Cycle	4	3	0	7			
Therapy Length							
1 day	0	0	0	0	6.65	4	0.156 NS
2 days	0	0	0	0			
3 days	1	0	0	1			
4 days	0	1	0	1			
5 days	31	9	22	31			
6 days	10	4	6	10			
7 days	0	0	0	0			
8 days	2	2	0	2			

## Discussion

It is crucial for children with OM and their parents/caregivers to have knowledge of chemotherapy-induced oral mucositis, preventive measures, and treatments for better outcomes. Self-management skills and awareness regarding OM can improve oral health using the APHO nursing intervention [8].

The present study is a prospective comparative study aimed at evaluating the effectiveness of a preventative oral care program in minimizing chemotherapy-induced oral mucositis in children with cancer. The study included 42 children aged six to 17 who were given an oral care protocol, including tooth brushing and mouthwash with chlorhexidine 0.2%, and compared with a control group. It supports the use of oral care guidelines in preventing pediatric cancer patients receiving chemotherapy from developing oral mucositis [9].

A randomized control trial supported another study in which the researcher found the highest incidence, pain, and severity of OM in the second week in patients undergoing chemo-radiation. The incidence of pain and severity of OM was reduced in the third and fourth weeks after using a sodium bicarbonate 5% mouthwash [10]. In the present study, chlorhexidine mouthwash along with easy-treat mouthwash was administered to the children twice a day for a week and had a drastic effect on OM. The severity and pain were reduced within a week.

A longitudinal study conducted by Yavuz and Bal Yilmaz in 2015 evaluated the effect of education regarding mouth care to pediatric cancer patients on the degree of OM. Sixteen samples were included in the age group of eight to 18 hospitalized in the oncology unit. The study results revealed a statistically significant difference between the degree of OM before and after education (p < 0.05), and the level of pain scores were also different before and after providing education (p < 0.05). After education, there was a positive and strong correlation between the degree of OM as well as pain score (p < 0.001). The severity of pain decreased when mouth care was performed daily [11].

This study revealed that the majority of samples belong to the first and second cycles of chemotherapy, meaning newly diagnosed children with cancer and their caregivers must be aware of the treatment and its side effects. It is essential to provide counseling before treatment to prevent OM and treat it after the occurrence.

Researchers have conducted studies to evaluate the effectiveness of various oral regimens, such as sodium bicarbonate, BG paint, aloe vera gel, oracef gel, candid mouth paint, and various mouthwash, such as normal saline wash, betadine gargles, chlorhexidine mouthwash [12], salt and soda mouthwash, and fluoride mouthwash. Some observational, comparative, and incidental studies have been conducted and found effective. However, these studies focus only on one aspect, whereas the present study focuses on a collaborative aspect. 

Limitation of the study

The limitation of the study was that it was conducted in a single center with a small sample group. The duration for follow-up was eight days, which may influence outcomes.

## Conclusions

The present study demonstrated the effectiveness of the intervention in improving the oral health of children undergoing chemotherapy and experiencing mucositis. These findings underscore the importance of preventing and managing oral mucositis in this population. Furthermore, the study highlights the potential benefits of APHO nursing intervention and emphasizes the value of collaborative approaches to improve patient outcomes and quality of life. The results of this study suggest that increased awareness and implementation of nursing interventions can contribute to better management and prevention of oral mucositis in pediatric cancer patients.
